# A new approach to near-surface positron annihilation analysis of ion irradiated ferritic alloys

**DOI:** 10.1039/d1na00394a

**Published:** 2021-09-03

**Authors:** Vladimír Kršjak, Petr Hruška, Jarmila Degmová, Stanislav Sojak, Pavol Noga, Tielong Shen, Veronika Sabelová, Werner Egger, Vladimír Slugeň

**Affiliations:** Slovak University of Technology in Bratislava, Faculty of Electrical Engineering and Information Technology, Institute of Nuclear and Physical Engineering Ilkovicova 3 Bratislava 81219 Slovakia jarmila.degmova@stuba.sk; Charles University, Faculty of Mathematics and Physics V Holesovickach 2 Prague 18000 Czech Republic; Slovak University of Technology in Bratislava, Faculty of Materials Science and Technology in Trnava, Advanced Technologies Research Institute Jana Bottu 2781/25 Trnava 91724 Slovakia; Chinese Academy of Sciences, Institute of Modern Physics Lanzhou Gansu 730000 China; Universität der Bundeswehr München, Institut für Angewandte Physik und Messtechnik Werner-Heisenberg-Weg 39 D-85577 Neubiberg Germany

## Abstract

The present work provides an innovative approach to the near-surface slow-positron-beam (SPB) study of structural materials exposed to ion-beam irradiation. This approach enables the use of variable-energy positron annihilation lifetime spectroscopy (PALS) to characterise a wide range of microstructural damage along the ion implantation profile. In a typical application of the SPB PALS technique, positron lifetime is used to provide qualitative information on the size of vacancy clusters as a function of the positron energy, *i.e.*, the probing depth of the spectrometer. This approach is limited to a certain defect concentration above which the positron lifetime gets saturated. In our experiments, we investigated the back-diffusion of positrons and their annihilation at the surface. The probability of such an event is characterised by the positron diffusion length, and it depends on the density of lattice defects, even in the saturation range of the positron lifetime. Until now, the back-diffusion experiments were reported only in connection with Doppler broadening spectroscopy (DBS) of positron-annihilation radiation. To verify the validity of the used approach, we compared the obtained results on helium-implanted Fe9Cr alloy and its oxide dispersion strengthened variant with the transmission electron microscopy and “conventional” slow positron DBS analysis.

## Abstract 2

Positron diffusion in helium-implanted Fe9Cr steel was investigated in a new approach revealing the role of microstructure in radiation embrittlement.

## Graphical abstract

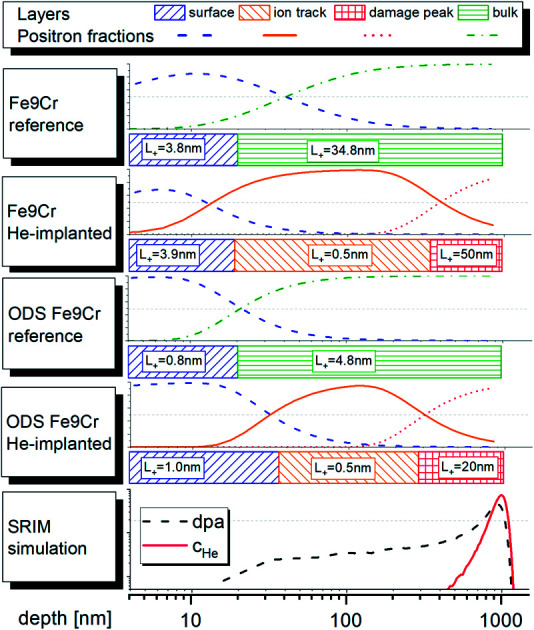

## 1. Introduction

Irradiation of bulk specimens in neutron radiation environments of various nuclear reactors inevitably leads to an induced activity of the tested samples. To minimise the handling of “hot” radioactive materials, research studies are either aimed at small-scale (miniaturised) samples^1^ or relatively low neutron fluencies.^2,3^ To experimentally simulate the neutron environment without the induced activity, neutrons can be effectively replaced in irradiation experiments by charged particles such as protons,^4^ alphas^5^ or self-ions,^6^ respectively. A standalone ion implantation experiment, or irradiations using a combination of two,^7^ or three^8^ different ions simultaneously, enables very efficient simulation of the environments of thermonuclear reactors, with high production rates of transmutation elements such as hydrogen or helium. However, high displacement damage rates of ion implantation in a narrow near-surface region limit the scientific value of ion implantation studies. The non-uniform damage profile requires investigation by depth-sensitive methods, enabling the probe of different regions within the Bragg curve. Typical experimental tools suitable for such studies are a quasi-non-destructive focused ion beam (FIB) lift-out technique followed by transmission electron microscopy (TEM) characterisation or a non-destructive slow positron beam (SPB) technique of positron annihilation spectroscopy (PAS).

Numerous studies have been published in the last two decades on SPB studies of ion-implanted samples.^9–12^ The experiments were focusing either on positron annihilation lifetime spectroscopy (PALS)^9^ or on measurements of the momentum distribution of electrons in a material^10–12^ using Doppler broadening spectroscopy (DBS) of the annihilation line. Particular attention was paid to study the helium behaviour in Fe-based materials due to its importance in nuclear fusion related research. PAS has proven to be an excellent tool for the characterisation of lattice imperfections in acting as sinks for radiation-induced defects and transmutation helium.^13,14^ The role of oxide precipitates in the radiation resistance of ferritic/martensitic steels has been investigated by both commonly used PAS techniques, PALS and DBS. There are, however, certain limitations of the applicability of PAS, particularly the positron lifetime technique used in the studies of materials with a high concentration of nano-features such as oxide-dispersion strengthened steels (ODS). The positron trapping in these materials is often saturated, *i.e.*, all positrons are trapped at defects and further introduction of defects (*e.g.*, by irradiation) does not lead to a clear response in positron lifetime spectra. However, positron back-diffusion measurements using slow (monoenergetic) positron beams have a higher sensitivity limit.^14^ Positrons implanted in the near-surface region of the studied sample have a certain probability of diffusing back and annihilating at the surface. Such annihilation can be clearly distinguished from the annihilation in the material bulk, and the probability of such an event depends on the density of lattice defects, even in the saturation range of the positron lifetime technique. The probability of the back diffusion to the surface is characterised by the positron diffusion length.^15^

The investigation of positron diffusion in ion-irradiated Fe-based alloys has been previously published in a few research studies.^9,16^ The experimental values of the positron diffusion length are commonly obtained using one-dimensional Doppler broadening spectra, which were collected as a function of the positron energy. To obtain a sufficient number of counts in the spectra in reasonable acquisition time, typically either a strong radioisotope positron source with a giga-Becquerel activity is required, or, alternatively, one can use large-scale facilities utilising intense gamma sources (either bremsstrahlung emitted from decelerating electrons or gamma released from nuclear reactions), producing electron–positron pairs. While the DBS technique is suitable for SPB studies and provides chemical information from the vicinity of the annihilation site, it does not provide qualitative information on the size of vacancy clusters, for instance. From this point of view, the positron lifetime technique is superior to the DBS technique. Nevertheless, a slow positron lifetime spectrometer requires precise timing of the pulses and much higher positron flux. This limits the choice of suitable positron sources to large-scale facilities utilising pair production by intense gamma sources.

The pulsed low energy positron system (PLEPS)^17^ installed at the high-intensity positron source NEPOMUC^18^ is one of few and the first facility of its kind, enabling precise positron lifetime measurements with variable-energy positron beam. This paper demonstrates the feasibility of using the lifetime spectra obtained at PLEPS or any other pulsed low-energy positron beam to determine positron diffusion length. The obtained results are compared to previous DBS measurements on the same helium-implanted materials.^16^ The present study complements and concludes decade-long research aimed at applying PAS techniques to improve the fundamental understanding of radiation tolerance of nano-oxide dispersion-strengthened steels.

## 2. Materials and methods

The present study was conducted on two Fe9Cr steels, namely Eurofer 97 and the ODS variant of this steel. The nominal chemical composition of Eurofer 97, as well as basic material characteristics, can be found in ref. 19. The ODS variant was prepared by mechanical alloying of the base steel powder prepared by inert gas atomisation with 0.3 wt% yttrium oxide powder.^20^

### 2.1 Ion implantations

**Fig. 1:**
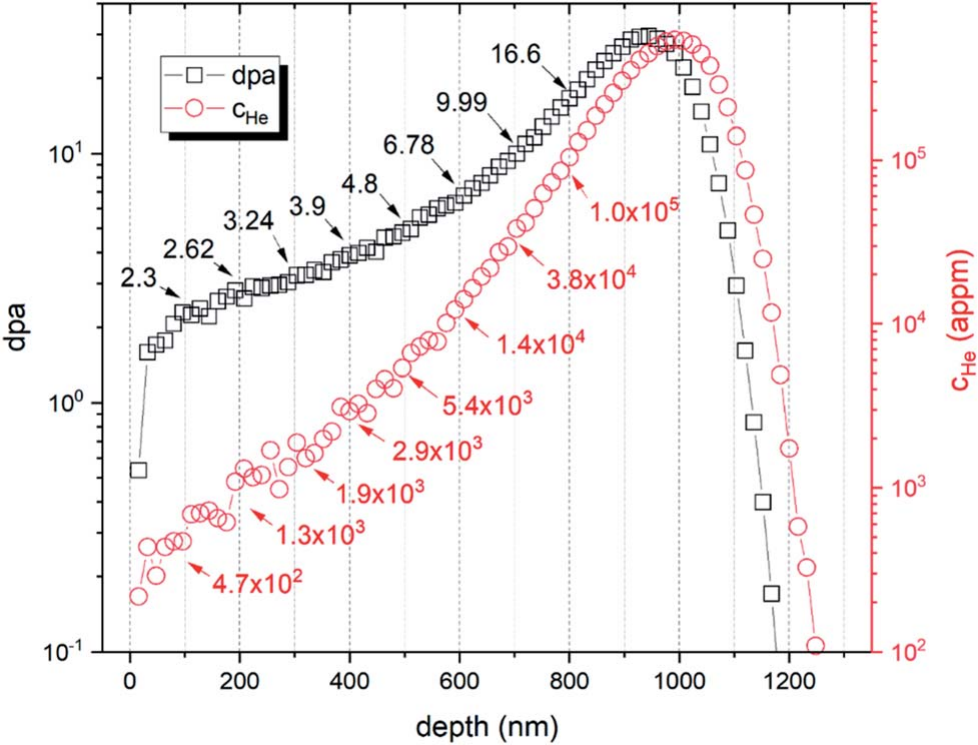
SRIM simulation of the ion implantation experiment showing displacement damage (dpa) and helium concentration (*c*_He_).

Samples were prepared by electrical discharge cutting and mechanical polishing, followed by electrochemical polishing in a solution of perchloric acid and acetic acid. To ensure the removal of the plastic deformation of the surface layer, introduced during the mechanical polishing process, >15 μm of the sample surface was electropolished out. Two samples of both investigated materials were implanted by 500 keV He^+^ ions with a fluence 10^22^ m^−2^ using the ion implanter at ATRI MTF STU.^21^ Irradiation temperature was kept near room temperature. The number of displacements per atom (dpa), calculated according to the NRT model^22^ and averaged over a 1.6 μm region, was 6.5, with a peak value of 29.5. According to SRIM^23^ simulation, the dpa peak was obtained in a depth of 950 nm, while the helium ion peak concentration was obtained in 1000 nm depth (Fig. 1).

### 2.2 Slow positron beam experiments

Two implanted samples, together with the reference samples, were investigated at the PLEPS facility.^17^ The investigation was performed within one week after the He^+^ implantations. During this period of time, the samples were kept in a dry sealed plastic container in order to minimise surface oxidation. Positron annihilation lifetime spectroscopy (PALS) measurements were performed using positron beam energies in the range of 0.5–18 keV. Given the density of the studied Fe9Cr alloys [7.9 g cm^−3^], this energy range approximately corresponds to a mean positron implantation depth in the range of 2–520 nm. The choice of the implantation peak being placed beyond the positron stopping maximum was due to our desire to focus on engineering-relevant irradiation conditions in terms of helium production rates. For each positron energy, at least 4 × 10^6^ annihilation events were collected, and the lifetime spectra were analysed in the LT10 program^24^ using a standard trapping model^19^ and a three-component decomposition. Unlike conventional lifetime spectra obtained using encapsulated radioisotope sources, the so-called source component, describing the positron annihilation in the source and its encapsulation, was not considered during the fitting.

The obtained positron lifetime results were compared to our earlier study of these materials using Doppler broadening spectroscopy (DBS). The DBS experiments, performed at the Aalto university's DBS facility, were reported in details in ref. 16 and 25. Although the DBS results were obtained for positron energies ranging between 0.5–36 keV, in this work, we use reference data for the same energy range that was used for PALS. It is important to note that the samples characterised by DBS were not electrochemically polished, and the surface was prepared only by mechanical polishing with silicon carbide paper followed by diamond paste with a final grade of 1 μm. This was considered in the analysis of the experimental data and the comparison with the PALS results.

The fundamental advantage of slow positron beam data lies in a depth-sensitive representation of the microstructure, which is enabled by the variable energy of the used positron probes. The mean implantation depth of positrons, z [nm] can be easily estimated for most metals using the incident positron energy *E* [keV] and the target materials density *ρ* [g cm^−3^] according to eqn (1).^26^1
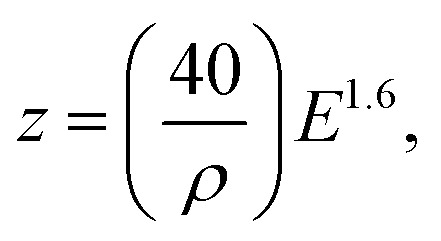


**Fig. 2:**
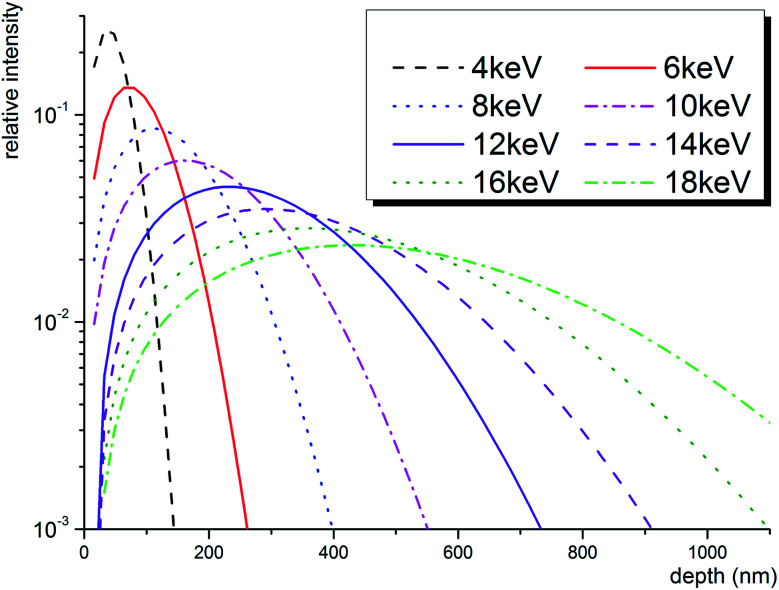
Theoretical positron stopping profiles in Fe-based alloy calculated using Makhovian distribution.^27^

In the case of the inhomogeneous depth distribution of microstructural defects, it is important to consider the whole stopping profile of monoenergetic positrons described by the Makhovian distribution.^27^ As illustrated in Fig. 2, this distribution broadens with increasing positron energy. While at low energies, the stopping profile is localised in a relatively narrow region (tens of nm to few hundreds of nm), higher energy positrons (≥12 keV) are distributed over a relatively wide region (several hundreds of nm). This needs to be considered in the interpretation of data obtained on ion-implanted specimens, also addressed in this study, whose depth distribution of radiation-induced defects follows the Bragg curve. More details on this issue can be found, for instance, in^10^.

The introduction of new vacancy-type defects in solids can be characterised by positron annihilation spectroscopy either in terms of an introduction of a new defect component(s) in positron lifetime spectrum (PALS measurements) or in terms of an increase of the line shape *S* parameter (DBS measurements).^2^ Positron lifetime is given by an overlap of the positron density and the local electron density at the annihilation site. *S* parameter characterises the Doppler broadening of the annihilation line due to the non-zero momentum of the annihilating electrons. Both quantities, therefore, carry information about the annihilation site, *i.e.*, local electron density and local electron momentum distribution, respectively. The average increase of positron lifetime or the increase of *S* parameter usually indicates enhanced trapping at defects in the studied material. In irradiation experiments with high production rates of helium, there appears, however, a competitive process to the increase of positron trapping. The reason is a fast migration of interstitial helium into vacancies, resulting in a decrease of both positron trapping and positron localisation at vacancy-type defects.

In a perfect lattice, all positrons annihilate in the free delocalised state. The introduction of defects into the perfect lattice leads to a localisation of positrons at these defects (introduction of new positron lifetime component) and a reduction of the free positron lifetime, as described by the simple trapping model, eqn (2).^19^2
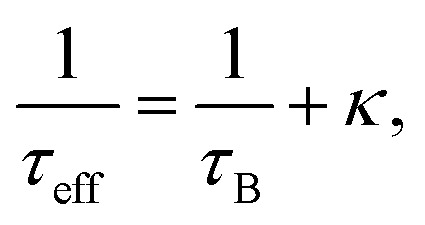
where *τ*_eff_ [s] is an experimentally obtained lifetime of a bulk-related free positron component (in addition to the lifetime of a defect-related component *τ*_2_), *τ*_B_ [s] is the lifetime in defect-free bulk. The positron trapping rate at defects, *κ* [s^−1^], is directly proportional to the volume density of the given type of defects. The proportionality constant, called the positron trapping coefficient *μ* [m^3^ s^−1^], has been recently experimentally obtained for nanometric helium bubbles in ref. 28.

When more than one type of defect is present in the material, it is reasonable to consider the evaluation of the mean positron lifetime (*τ*_mean_), rather than the lifetimes and intensities of the individual components of the spectra. *τ*_mean_ is defined as a weighted average of positron lifetime components. According to the simple trapping model, with increasing concentration of defects, the positron trapping at defects is enhanced, the effective lifetime *τ*_eff_ is reduced by the trapping rate, while the mean positron lifetime *τ*_mean_ increases.

It is important to note that the trapping rate is proportional to the density of traps only in so-called transition-limited positron trapping, which assumes a small size of positron traps with a spatial extent much less than the positron de Broglie (thermal) wavelength (∼few nm).^19^ In such case of homogeneously distributed small defects, the positron diffusion constant *D*_+_ is not a function of space coordinates, and the effective positron lifetime *τ*_eff_ is proportional to the effective positron diffusion length in the given material (eqn (3)).3
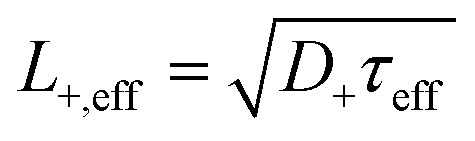


Using the above two equations, one can express the positron trapping rate at defects [s^−1^] *via* positron diffusion length as follows:4
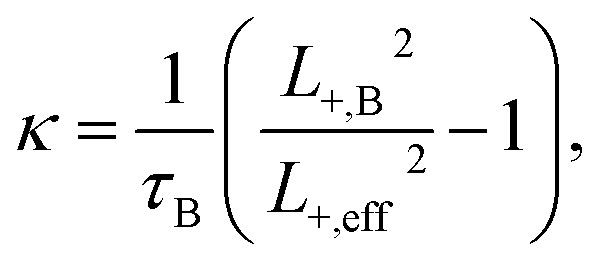
where *L*_+,B_ is the positron diffusion length in defect-free bulk. The positron trapping rate can be obtained by eqn (4) even in the case of saturated positron trapping when the positron lifetime does not reflect the increased concentration of defects anymore. This approach is very beneficial in studies of tempered martensitic steels irradiated to high displacement damage levels. The experimental values of the effective positron diffusion length can be conventionally obtained from slow positron beam-based DBS measurements. The *S* parameter depth profiles can be analysed using the VEPFIT code developed by van Veen *et al.*,^29^ which numerically solves the positron diffusion equation.

As mentioned above, the mean positron lifetime (measured by PALS) and the *S* parameter (measured by DBS) carry information about the local electronic structure at the annihilation site. The total *S* parameter can be, similarly to the eqn (2), expressed as a superposition of (generally unknown) partial *S* parameters,††While the positron lifetime values obtained from the deconvolution of the PALS spectra are commonly attributed to particular bulk materials or particular lattice defects, the experimental DBS spectrum cannot be “deconvoluted” into bulk and defect component in such manner. Yet, it is clear that the annihilation of free positrons and positrons trapped at defects results in different shape of the broadened DBS profiles. corresponding to the annihilation of free positrons and positrons trapped at defects, respectively. Despite this analogy, the positron lifetime depth profiles were, to our knowledge, never reported to be used in data treatment using VEPFIT code. To justify and support this procedure and compare the results of the slow positron beam measurements using PALS and DBS techniques, we need to describe the behaviour of positrons near the interface of two layers, which are characterised by different *S* parameters and mean positron lifetimes. For each positron energy, the distribution of depths, where positrons annihilate, is broadened by implantation profile, described by Makhovian distribution, and following diffusion of thermalised positrons, characterised by the effective positron diffusion length. As a result, the experimentally obtained mean lifetime and *S* parameter values are given by the superposition of mean lifetimes *τ*_mean,1_ and *τ*_mean,2_, and *S* parameters *S*_1_ and *S*_2_, respectively.

5 *τ*_mean_ = *n*_1_*τ*_mean,1_ + *n*_2_*τ*_mean,2_

6 *S* = *n*_1_*S*_1_ + *n*_2_*S*_2_

Fractions *n*_1_ and *n*_2_ of positron annihilation in layer 1 or layer 2, respectively, are the same for the mean positron lifetime as well as for the *S* parameter. Therefore, both parameters follow the same phenomenon, which is comprehended by the VEPFIT program very well. In this case, applying the VEPFIT analysis, usually used for the analysis of *S*(*E*) curves obtained by DBS, to the analysis of *τ*_mean_(*E*) measured at PLEPS is justified. There is, nevertheless, a limit in the applicability of the presented approach. Unlike the *S*-parameter and defect positron lifetime components, which are inherent to a given layer in a multilayer system, the free-positron lifetime component and therefore the positron mean-lifetime are not inherent to a single layer and depend also on the properties (*e.g.*, defect concentrations) of adjacent layers. This means that eqn (5). can be used as long as the contribution of free positrons to positron mean-lifetime is insignificant as in the case of saturated trapping. Although this seems to be a strong condition, one must keep in mind that all PALS experiments on realistic structural materials, such as steels, exposed to harsh radiation conditions essentially lead to a saturated or a near-saturated positron trapping.

### 2.3 TEM characterisation

The cross-sectional TEM samples of the irradiated steels were prepared using the focused ion beam (FIB) lift-out techniques in the FEI Helios Nanolab system, with a Ga acceleration voltage in the range from 1 kV to 30 kV. In the next step, a cleaning process to remove the FIB damaged layer was performed by low energy (Ga) ion milling. Microstructural characterisation was performed using FEI Tecnai F20 TEM, with an accelerating voltage of 200 kV. TEM thin foils, mounted on a double-tilt stage, were tilted away from the zone axis for emphasising the bubble contrast. The thickness of each lamella was obtained by TEM and varied between 65 and 75 nm. Under-focused bright-field images were performed for characterising He bubble distributions. In this work, bright-field TEM images were taken at an exposure time of 2 s by a CCD camera with 4008 × 2672 pixels.

## 3. Results and discussion

To complement and verify the proposed methodology based on slow positron beam experiments, cross-sectional TEM observations have been performed. The obtained TEM data provided the initial guess values of the layer boundaries as well as the qualitative characterisation of helium bubbles in the range of irradiation conditions, where the helium production is in the same order of magnitude as the concentrations expected in the spallation neutron target.

### 3.1 TEM characterisation

**Fig. 3:**
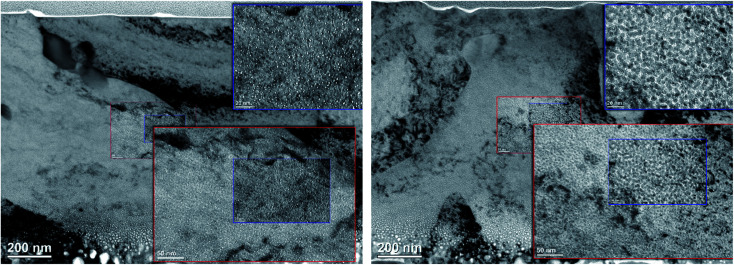
Cross-sectional TEM image of the investigated Fe9Cr alloy (left) and its ODS variant (right). The upper right insets correspond to a region 450–580 nm below the surface.

**Fig. 4:**
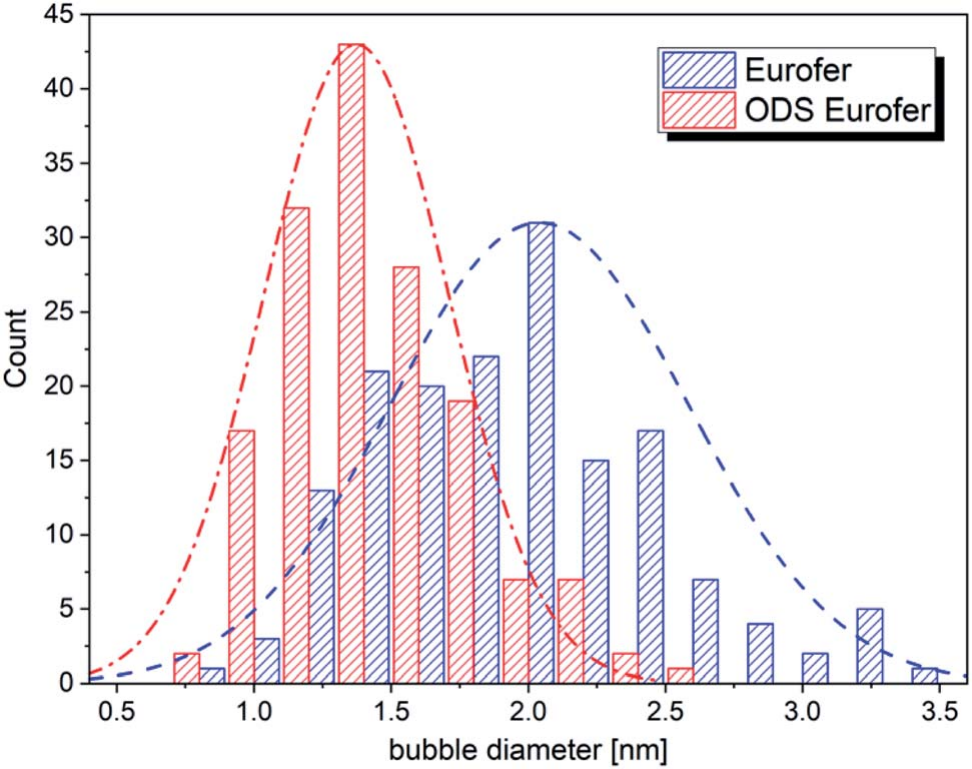
The size distribution of helium bubbles in the studied materials, corresponding to the magnified insets in Fig. 3.

The cross-sectional TEM observation enabled to identify the region with fully grown helium bubbles and to provide a qualitative analysis in terms of their size distribution. For both materials, TEM-resolvable (>1 nm) He bubbles were observed in the region of >300 nm below the surface, which corresponds to displacement damage of >2.5 dpa and helium concentration (*c*_He_) of >1000 appm. Due to limited spatial resolution, TEM analysis did not indicate any major enhancement of radiation resistance for ODS steel in these “low” irradiation conditions. A detailed TEM analysis was performed in the region with substantially higher helium concentration. Fig. 3 shows a cross-sectional TEM image of the two studied materials, with magnified insets corresponding to 450–580 nm, *i.e.*, 3600–10 800 appm He, or 4.0–5.9 dpa, respectively. The obtained size distribution of helium bubbles is shown in the histogram in Fig. 4.

As can be seen in Fig. 4, the average size of helium bubbles is significantly lower (<1.5 nm) in the ODS alloy in comparison to non-ODS alloy (∼2 nm). With increasing depth and with approaching displacement damage and helium concentration peaks, the size of bubbles grows significantly (well above 5 nm), but without an obvious difference between the two studied materials. The possible reason for the similarity in the microstructural evolution is the increasing dominance of bubbles in the sinking of new vacancies and helium atoms,^30^ as well as in the coalescence of the bubbles.^5^

### 3.2 PAS characterisation

#### 3.2.1 Positron annihilation in inhomogeneous samples

Previous PAS studies on high-temperature irradiation experiments with material microstructure containing large voids or bubbles (>5 nm)^30^ concluded that positron trapping in such case is diffusion-limited rather than transition-limited. For very large metallic voids with a radius greater than the positron thermal wavelength, the positron trapping is determined by the absorption of the incident positron wave at the void boundary.^19^ Strong trapping at large cavities depletes the positron flux in the vicinity of the void, and its rate is not necessarily proportional to the positron diffusion length as described by eqn (3). This has a direct implication for the present study, where different stages of microstructural evolution are investigated, ranging from a high density of small defects (helium-vacancy clusters) to large bubbles grown by coalescence.^5^ Our previous study on PAS investigation of f/m steels irradiated in spallation neutron target suggested that at a certain level of microstructural damage (in terms of helium bubble sizes), the increase of positron lifetime due to the growth of helium bubbles changes its trend towards saturation. This was explained by the coalescence of bubbles and the increase of positron mean free path.^31^

The present study, which proposes a new approach to obtain the effective positron diffusion length, builds on the well-known correlation between the mean positron lifetime obtained from PALS measurements and the line-shape *S* parameter obtained from DBS characterisation. The often nearly linear correlation between these two complementary PAS parameters has been reported in numerous papers in the past, including our study on helium embrittlement of f/m steels.^32–34^

The application of slow positron beam techniques, or depth-sensitive analytical techniques in general, on the characterisation experiments of ion-implanted samples is naturally facing the problem with the nonuniform damage profile. In the case of positron annihilation spectroscopy, the eventual need for a shift from transition-limited to diffusion-limited trapping may lead to some unexpected outcomes. In the first case, diffusion coefficient *D*_+_ is a constant and the effective positron lifetime is bound with diffusion length by eqn (3). As described above, increasing mean lifetime suggests enhanced trapping at defects, caused either by an increased concentration or increased size of defects. At the same time, the effective lifetime decreases due to enhanced trapping, and, according to eqn (3), the diffusion length decreases as well. In the second case (diffusion-limited trapping), the positron density becomes a function of spatial co-ordinates, since positron density is depleted in the vicinity of defects. In contrast, in the case of transition-limited trapping, positron density is independent on spatial co-ordinates (it depends on time only). For a constant *D*_+_, a positron diffuses on average for a time *τ*_f_ until it either annihilates as a free positron or is trapped in a defect. The trapping probability is given by the trapping rate *κ*, only.

Let us consider two hypothetical scenarios of structural materials, such as steel, exposed to a harsh radiation environment. In the first scenario, the irradiation experiment leads to a homogeneity in microstructure, which contains a high density of relatively shallow traps (“small” helium-filled vacancy clusters). In the second one, the irradiated microstructure is inhomogeneous and contains a lower density of deep positron traps such as “large” voids or bubbles. Comparing to the unirradiated material, the mean lifetime of positrons, as well as the positron trapping rate, will likely be higher in the irradiated materials in both scenarios. The effective positron diffusion length *L*_+,eff_, on the other hand, does not have to follow the same trend in the two scenarios. In the first scenario, *L*_+,eff_ decreases with the increase of positron trapping, *i.e.*, with the mean positron lifetime. A plausible outcome of the second scenario is that all three parameters, *i.e.*, mean positron lifetime, positron trapping rate and the effective diffusion length, will increase.

#### 3.2.2 Positron lifetime characteristics

Positron lifetime spectra of all studied materials were analysed using the three-state trapping model, where the first component corresponds to free positrons (reduced bulk lifetime *τ*_1_ < 110 ps), second component describes short-lifetime traps such as dislocations and/or small vacancy clusters (*τ*_2_ ∼ 160–250 ps), while the third lifetime component is attributed to large vacancy agglomerations (*τ*_3_ > 400 ps).^30^ Typical values obtained for the reference samples of the two studied materials from the slow positron beam lifetime spectra decomposition are shown in Table 1. Positron energies 4 and 18 keV represent roughly the ∼50 and ∼500 nm region below the surface. It is important to note that the contribution of free positrons, represented by reduced bulk lifetime *τ*_1_, was relatively low throughout all lifetime spectra presented here. In fact, the maximum intensity of the first component was 38.3% with an average value of ∼19.6 ± 6% and in both studied materials. Taking into account the average value of the reduced bulk lifetime being 32.1 ± 9.9 ps (*i.e.*, ≪ 110 ps), we can consider all investigated microstructures as being in a near-saturation state of positron trapping. In such conditions, the contribution of free positrons to the positron mean lifetime is rather low, and the eqn (5) is applicable.

**Table 1:** Example of data obtained from the decomposition of the positron lifetime spectra obtained on reference materials in different depths

*E* [keV]	Depth [nm]	*τ*_1_ [ps]	*I*_1_ [%]	*τ*_2_ [ps]	*I*_2_ [%]	*τ*_3_ [ps]	*τ*_AVG_ [ps]	Fit's variance
**Fe9Cr**								
4	50	54	38.3	230	41.0	446	207.0	1.06
18	500	35	12.9	158	84.0	520	153.3	1.02

**ODS Fe9Cr**								
4	50	42	26.0	220	59.0	450	208.4	1.07
18	500	33	19.1	215	78.0	550	189.6	1.12

**Fig. 5:**
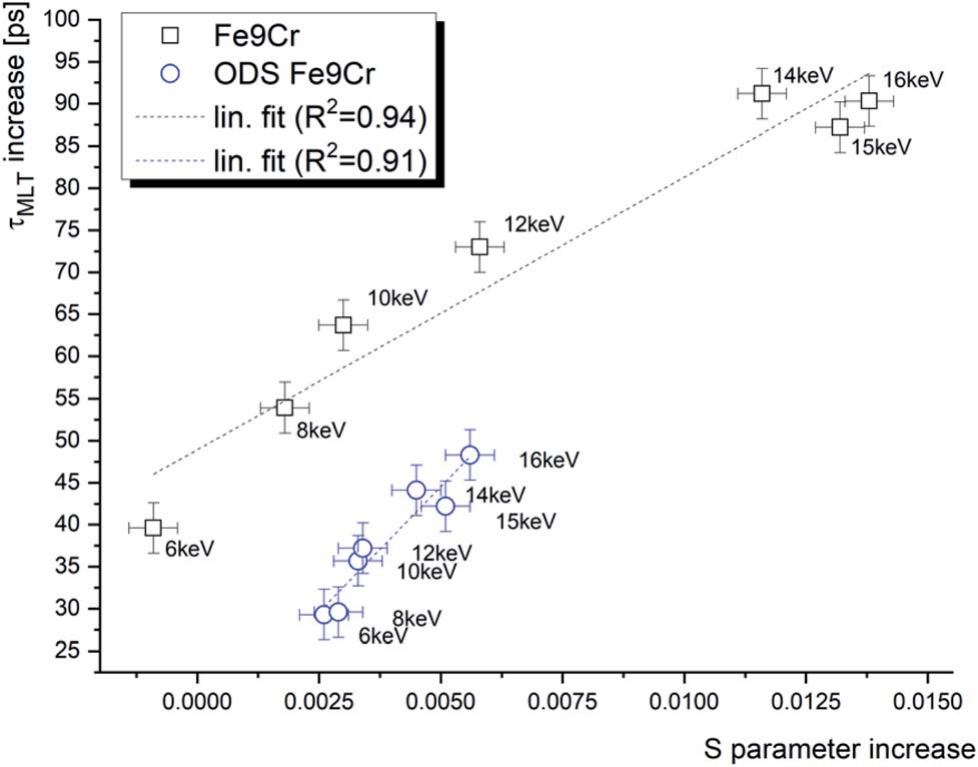
The positron mean lifetime (*τ*_MLT_) as a function of the *S* parameter in helium-implanted f/m steel for various positron energies.

In our recent work,^16^ we reported slow positron DBS characterisation of the same materials and implantation conditions. Fig. 5 shows the increase of the positron mean lifetime (*τ*_MLT_) after the implantation as a function of the *S* parameter increase in terms of absolute values. The reference values considered are the *S*-parameter and *τ*_MLT_ obtained for the un-implanted samples of the studied materials using the same positron beam energy. One can see a nearly linear dependence of the two parameters. A similar observation was reported in our previous work on f/m steels irradiated in a spallation neutron target with relatively high He production rates.^33^ This behaviour supports the assumption that the positron diffusion equation can be used to describe the microstructural damage in the same manner for both techniques utilising slow positrons. In other words, it justifies the analysis of the positron lifetime depth profiles using the VEPFIT software, which was, to our best knowledge, not reported in the literature until now. The slightly different slopes of the two data sets in Fig. 5 are due to different trapping rates of positrons in the two studied materials.

**Fig. 6:**
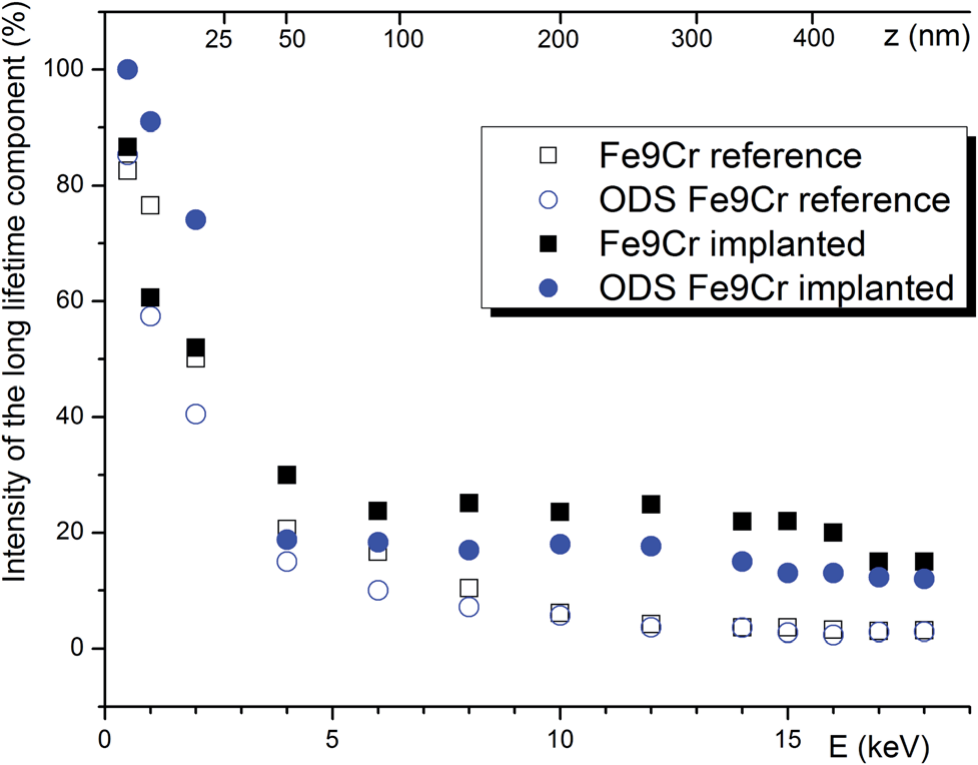
The intensity of the component(s) with positron lifetime > 260 ps. For the plotted data, a conservative estimate of the uncertainty is ± 5%.

The effect of positrons diffusing back to the surface was clearly observed in long lifetime components, whose intensities and lifetimes increase dramatically towards the surface. As can be seen in Fig. 6, the trapping at the surface starts to dominate the positron annihilation for energies below 5 keV. Using a conservative approach, we decided that the material bulk (*i.e.*, region of negligible surface effect) is probed by positrons with energies above 8 keV. Since the chemical composition, as well as the density of the two studied materials, is very similar, the surface effect is practically identical for both studied materials. After helium implantations, the bulk value of the intensity of the long defect component increases by 16.3% or 10.9% for the Fe9Cr and ODS Fe9Cr, respectively. This indicates the presence of new open volume defects produced during the irradiation.

**Fig. 7:**
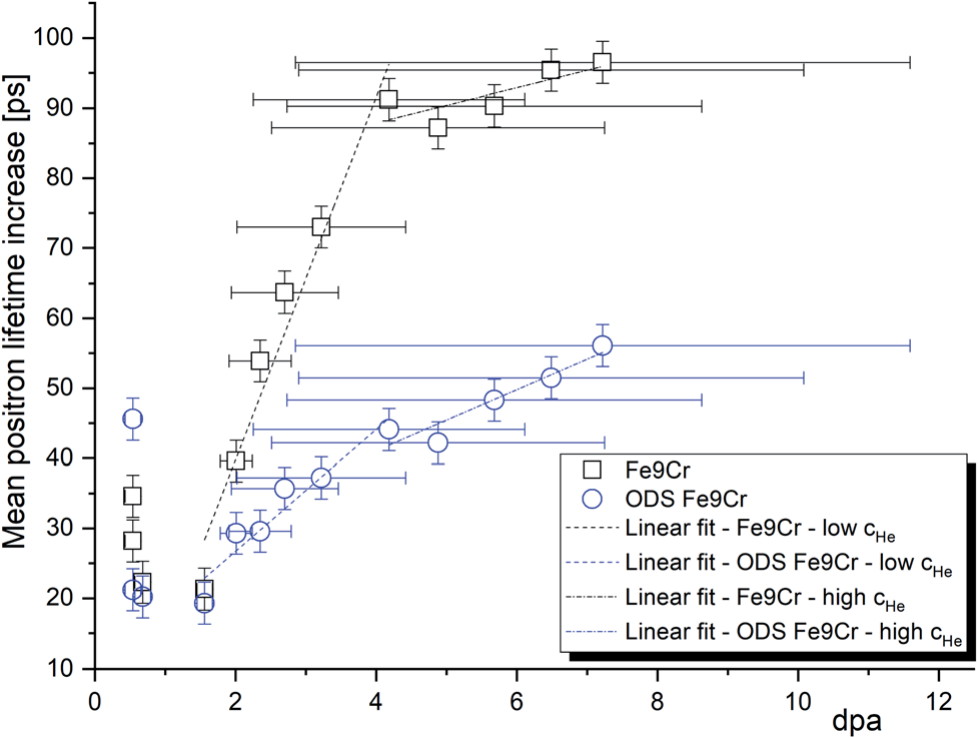
The increase of the positron mean lifetime after helium implantation in the Fe9Cr steel and its ODS variant. The error bars on the *x* (dpa) axis were obtained as the standard deviation of the Gaussian-like profiles.

From the materials perspective and their performance under irradiation, it is interesting to compare the two materials with respect to the mean positron lifetime profile and displacement damage (dpa). The mean positron lifetime, as the statistically most reliable parameter, is virtually independent of the fitting procedure. A lifetime spectrum represented by the positron mean lifetime is similar to a fitting of the spectra by a single exponential function. It is, therefore, reasonable to compare the material performance in a radiation environment using this parameter. As shown in Fig. 7, for both materials, the positron lifetime significantly increases after the implantation, but the increase is much more pronounced in the conventional (non-ODS) f/m steel. This is in excellent agreement with the TEM analysis shown in Fig. 4, and it demonstrates the well-known improvement of the radiation resistance of nanostructured f/m steels. It is reasonable to assume that the high density of sub-granular interfaces is acting not only as recombination centres for radiation-induced defects but also as sinks for (transmutation) helium suppressing the helium bubble swelling.

#### 3.2.3 VEPFIT analysis

In addition to direct assessment of positron lifetime data with respect to the irradiation conditions (dpa, *c*_He_), they were evaluated with respect to the diffusion length. This was performed using the VEPFIT code, which numerically solves the positron diffusion equation.^29^ As input data, positron mean lifetime [ns] attributed to given energy of the beam [keV] was used. The justification for this approach is provided earlier in this paper. The VEPFIT analysis (Table 2) of the mean positron lifetime profiles was performed by considering previous results on DBS analysis^16^ and similar published experiments.^35^ The reference value of the positron diffusion length in bulk Fe9Cr was 50 nm, as reported for the same material in ref. 35. This value was treated as a free parameter of the second layer in the two-layers fitting procedure. The first layer was attributed to surface oxide, which was expected to be very narrow compared to our previous experiments on mechanically polished samples.^16^ Fitting was performed in several steps with the aim of obtaining physically meaningful values and the best possible chi-squared value. It is important to note that, leaving too many free non-linear parameters in the fitting procedure occasionally resulted in an unstable fit. Consequently, some of the parameters must have been fixed during the fitting. By varying the values of the fixed parameters, we were looking for best achievable goodness-of-fit. The obtained results were considered physically meaningful when: (a) any obtained diffusion length was shorter than the diffusion length in defect-free iron; (b) the diffusion length in ODS material was shorter than in non-ODS material and (c) the obtained diffusion lengths were in a reasonable agreement with the values reported in the literature (*e.g.* ref. 35). The finally obtained values of positron diffusion lengths were 34.8 nm and 4.8 nm, for the Fe9Cr and its ODS variant, respectively. This is in excellent agreement with the previously reported results^16^ from DBS experiments (35 and 5 nm, corresponding to the “bulk layer” (layer 3) of the Fe9Cr steel and its ODS variant, respectively). In both materials, the surface oxide layer was 20 nm thick with a positron diffusion length below 4 nm. The best-achieved chi-squared values were 65.6 and 84.1, respectively, which is a reasonably good result, considering the number and scattering of data points. These results confirm a much higher density of positron traps, expected in the ODS steel compared to its non-ODS variant. These traps are considered to be acting as recombination centres for radiation-induced defects during the ion implantation and likely also as sinks for helium atoms. Note that surface preparation of samples in the previously published report^16^ was different and an extra strain-affected layer, introduced by mechanical polishing, was considered and confirmed by the experiment. The layer corresponding to the material bulk in this study was the second layer, while the “bulk layer” in the previously published study was the layer 3.

**Table 2:** Results of the VEPFIT analysis^a^

	PALS (this study)				DBS (adopted from ref. 16)			
Material	Fe9Cr		ODS Fe9Cr		Fe9Cr		ODS Fe9Cr	
Sample (reference/implanted)	Ref.	Impl.	Ref.	Impl.	Ref.	Impl.	Ref.	Impl.
**Fitting parameter *L***_**+**_ **[nm]**								
Layer 1	3.77 ± 0.19	3.94 ± 0.04	0.77 ± 0.04	1.00 ± 0.01	2.26 ± 0.37	4.87 ± 0.40	4.36 ± 0.47	1.68 ± 0.12
Layer 2	*34.8* ± 0.69	0.49 ± 5.37	*4.84* ± 0.29	**0.5**	**10**	**50**	**3.5**	**20**
Layer 3		**50**		**20**	***35***	**35**	***5***	**5**

**Fitting parameter – layer boundary [nm]**								
Layer 1	**20**	19.21 ± 5.61	**20**	36.86 ± 15.78	224.95 ± 12.25	399.1 ± 5.59	287.3 ± 13.29	531.6 ± 11.02
Layer 2		351.7 ± 6.83		293.6 ± 5.99	1376.41 ± 13.90	2055.9 ± 9.84	1209.8 ± 18.58	2017.0 ± 26.58

**Fitting parameters**								
	***τ***_**mean**_ **[ns]**				***S*-parameter**			
Layer 1	0.2812 ± 0.0010	0.4438 ± 0.0050	0.3204 ± 0.0012	0.45905 ± 0.0003	0.46 016 ± 0.0003	0.45608 ± 0.0003	0.45987 ± 0.0003	
Layer 2	0.1527 ± 0.0004	0.2201 ± 0.0005	0.1896 ± 0.0003	215.7 ± 0.0006	0.46 751 ± 0.0002	0.50 006 ± 0.0004	0.46450 ± 0.0003	0.48267 ± 0.0003
Layer 3		0.2650 ± 0.0009		250.2 ± 0.0007	0.43 949 ± 0.0006	0.39 292 ± 0.0018	0.44 672 ± 0.0005	0.50 149 ± 0.0029

a **Bold** = fixed values; in order to reduce the degrees of freedom and to obtain physically meaningful results, the highlighted parameters were fixed in the fitting procedure. *Italics* = reference materials bulk values.

The VEPFIT analysis of the implanted samples was performed using a three-layer model. The first layer was considered to be identical with layer 1 in un-implanted samples and its upper boundary was found to be 19.2 nm and 36.9 nm, respectively. The second layer with a very short diffusion length (0.5 nm) was attributed to a region with relatively high displacement damage and negligible He concentration. The upper boundary of this layer was found to be 351.7 nm and 293.6 nm, respectively. According to the SRIM implantation profile, this corresponds to ∼3.5 dpa and ∼1500 appm He. These values are reasonably close to the displacement damage and helium concentration of f/m steels irradiated in spallation neutron target, investigated by PAS techniques at the Paul Scherrer Institute.^31^ Although the irradiation temperature in spallation neutron targets is typically higher than in the presented experiment, in both cases, the microstructure of irradiated f/m steel leads to a (nearly) saturated positron trapping.^33^ This explains the very short positron diffusion length as well as a very short positron bulk lifetime component. The diffusion length of positron in the third layer, starting at implantation depth > 300 nm was found to be *L*_+_ = 50 nm and *L*_+_ = 20 nm for Fe9Cr steel and the ODS Fe9Cr, respectively. These values are higher than the diffusion length in the unirradiated bulk. Based on the TEM results and the discussion in chapter 4.2.1, we assume that the positron trapping undergoes a transition to a diffusion-limited regime between the interface of layers 1 and 2 and the displacement damage peak, most likely in the region 600 ± 100 nm. This explains the unexpected increase in positron diffusion length along with the increase of positron mean lifetime between the reference and the irradiated condition (layer 3) of both studied materials.

The present results demonstrate both the effect of displacement damage and the effect of inert gas implanted in the microstructure. From the theoretical work of Troev *et al.*,^36^ it is known that the presence of helium can counterbalance the increase of positron trapping due to new radiation-induced defects. While helium-to-vacancy ratio close to 1 was obtained by theoretical^37^ and experimental^38^ methods for small vacancy clusters, a significantly lower ratio of helium is expected in large cavities. It is reasonable to assume that helium bubbles and large nanometric voids do not dramatically reduce the depth of the potential well for positrons. As a consequence, strong trapping at these cavities depletes the positron flux in their vicinity, and the positron trapping rate is not proportional to the positron diffusion length according to eqn (3). We assume that this scenario takes place at helium concentration > 10 000 appm, *i.e.*, in depth higher than ∼600 nm in the samples studied in this work.

Our results show that the positron mean lifetime is very similar for the two studied materials after helium implantation, despite very different initial values. This, however, cannot be interpreted as the same performance under irradiation for two reasons. Firstly, the effect of helium on the reduction of positron trapping at small vacancy clusters is competing here with the effect of newly created vacancies, increasing the trapping. The qualitative and quantitative information on vacancy-type defects cannot be, therefore, easily obtained from the PALS measurements. The second reason is the limited effect of the vacancy-type defects on the performance of the irradiated material. Since the PAS analysis is not sensitive to interstitial-type defects, the comparison of PAS results with physical and mechanical properties will always be subject to error.

**Fig. 8:**
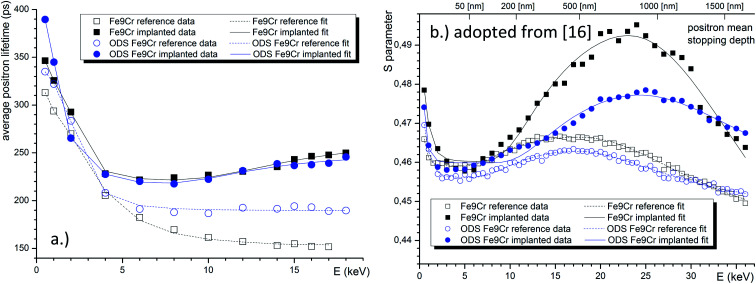
VEPFIT analysis of the positron lifetime depth profiles of the two studied materials in the pristine and implanted conditions (a). Analogous analysis of the Doppler depth profiles, adopted from our previous work (b).^16^

The fitted curve is shown together with the experimental lifetime values in Fig. 8. When considering the material bulk, as argued above, to be probed by positrons with *E* > 8 keV, we can conclude that the positron lifetime increased after implantation by 81.4 ps and 43.1 ps, for the Fe9Cr and its ODS variant, respectively. This is evidence of the presence of a higher density of recombination centres in the oxide dispersion strengthened steel, suppressing the formation of large defect agglomerations. Fig. 8b confirms the anticipated presence of an extra layer in the reference DBS samples, corresponding to microstructural defects introduced by mechanical polishing of these samples. This is indicated by the *S*-parameter peak in the region ∼500 nm. A similar feature, *i.e.*, a peak in the mean lifetime values, was not observed in the PALS data. This points out the importance of the electropolishing step in the surface treatment of the samples.

**Fig. 9:**
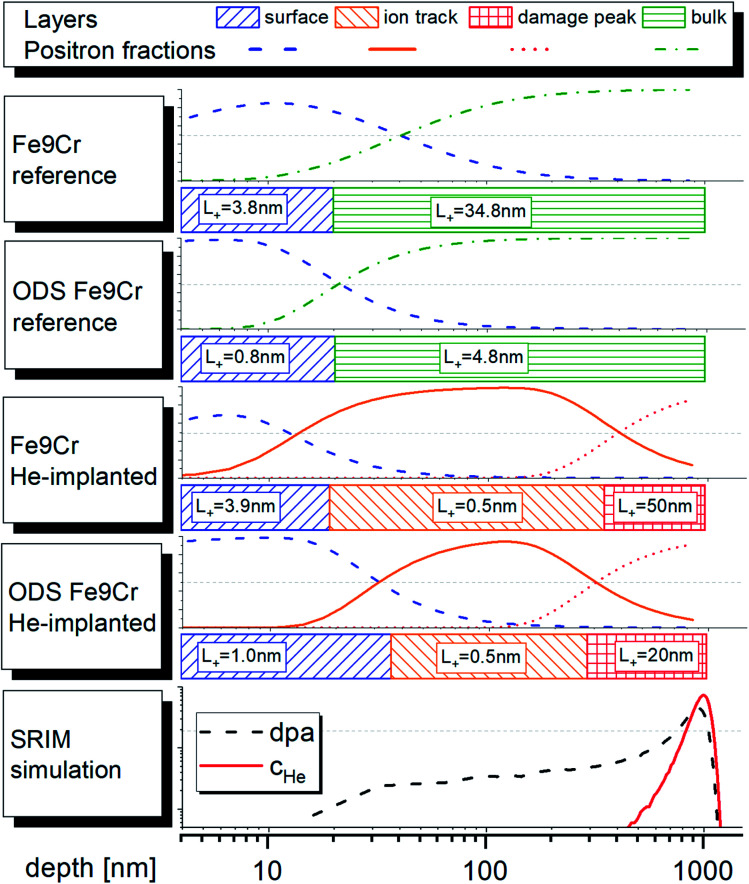
Schematic representation of the results of the VEPFIT analysis and the normalised distribution of the dpa and c_He_ profiles (area under the curve is unity), as obtained from SRIM. The fractions of positron annihilating in the individual layers are shown for all samples. The vertical axis of the fractions represents a linear scale from 0 to 100%.

The schematic representation of the fitting models used in the VEPFIT analysis of all studied samples is shown in Fig. 9. The figure also shows the contribution (fractions) of positron annihilating in the individual layers as a function of depth. Note that thermal and epithermal positrons are not shown due to their small to negligible contribution.

## Conclusions

The present work provides an innovative approach to the experimental study of inhomogeneous radiation damage in ion beam modified materials. Particular focus is placed on the investigation of materials bombarded with ions of an inert gas such as helium, which stabilises radiation-induced vacancy-type defects and represents a significant challenge in developing materials for many future nuclear installations.

We showed that the positron diffusion length, which is an important parameter reflecting the microstructural damage of materials, can be obtained directly from the mean positron lifetime depth profiles. The results acquired from the VEPFIT analysis of the positron mean lifetime are in excellent agreement with the conventional approach based on the fitting of the Doppler broadening *S*-parameter depth profiles. Unlike conventional slow positron lifetime studies, the presented methodology enables the investigation of radiation-induced defects in the saturation of positron trapping when the missing contribution of free positrons excludes the quantitative characterization of lattice defects. By considering positron diffusion, we were able to extrapolate the meaningful outcome of the positron lifetime data towards the peak of the concentration of the defects produced by ion implantation. The reported methodology is particularly interesting for studies of complex materials like nanostructured alloys in which the positron trapping gets easily saturated.

The studied Fe9Cr f/m steel and its ODS variant showed both a significant reduction of the positron diffusion length in the helium implanted region, followed by a substantial increase in the helium peak region. This increase was attributed to the growth of helium bubbles, likely by coalescence, resulting in large volumetric defects which act as strong positron traps and lead to a diffusion-limited positron trapping.

The enhanced radiation tolerance of the ODS steel, indicated by the size distribution of large TEM-visible cavities, was also observed in the sub-nm size range by positron annihilation lifetime spectroscopy *via* a less pronounced increase of the positron lifetime. On the other hand, no significant indication of improved radiation tolerance has been observed for either of the materials at high displacement damage and helium concentration levels.

## Conflicts of interest

There are no conflicts to declare.

## Supplementary Material
